# Paving the way to nanoionics: atomic origin of barriers for ionic transport through interfaces

**DOI:** 10.1038/srep17229

**Published:** 2015-12-17

**Authors:** M. A. Frechero, M. Rocci, G. Sánchez-Santolino, Amit Kumar, J. Salafranca, Rainer Schmidt, M. R. Díaz-Guillén, O. J. Durá, A. Rivera-Calzada, R. Mishra, Stephen Jesse, S. T. Pantelides, Sergei V. Kalinin, M. Varela, S. J. Pennycook, J. Santamaria, C. Leon

**Affiliations:** 1 GFMC, Departamento de Física Aplicada III, Facultad de Física, Universidad Complutense de Madrid, Campus Moncloa, 28040 Madrid, Spain; 2 Materials Science & Technology Division, Oak Ridge, TN 37831, USA; 3 Center for Nanophase Materials Sciences, Oak Ridge, TN 37831, USA; 4 Department of Physics and Astronomy, Vanderbilt University, Nashville, TN 37235, USA; 5 Department of Materials Science and Engineering, The University of Tennessee, Knoxville, TN 37996, USA; 6 Departamento de Química-INQUISUR,Universidad Nacional del Sur, Av. Alem 1253, Av. Alem 1253,, 8000 Bahía Blanca, Argentina

## Abstract

The blocking of ion transport at interfaces strongly limits the performance of electrochemical nanodevices for energy applications. The barrier is believed to arise from space-charge regions generated by mobile ions by analogy to semiconductor junctions. Here we show that something different is at play by studying ion transport in a bicrystal of yttria (9% mol) stabilized zirconia (YSZ), an emblematic oxide ion conductor. Aberration-corrected scanning transmission electron microscopy (STEM) provides structure and composition at atomic resolution, with the sensitivity to directly reveal the oxygen ion profile. We find that Y segregates to the grain boundary at Zr sites, together with a depletion of oxygen that is confined to a small length scale of around 0.5 nm. Contrary to the main thesis of the space-charge model, there exists no evidence of a long-range O vacancy depletion layer. Combining ion transport measurements across a single grain boundary by nanoscale electrochemical strain microscopy (ESM), broadband dielectric spectroscopy measurements, and density functional calculations, we show that grain-boundary-induced electronic states act as acceptors, resulting in a negatively charged core. Besides the possible effect of the modified chemical bonding, this negative charge gives rise to an additional barrier for ion transport at the grain boundary.

Solid-state electrochemical devices such as batteries and fuel cells are the key to commercially viable low power energy generation and storage^1^,^2^,^3^,^4^. Their operation relies on ion transport through solid electrolytes that are usually in polycrystalline form, and thus the performance is strongly affected by the blocking of ions at grain boundaries. Ion transport through grain boundaries is even more relevant in the case of nanoionics^5^,^6^,^7^,^8^, due to the increasing influence of interfaces compared to the bulk when decreasing the size of the device. However, the origin of the barriers for ionic transport at grain boundaries is not well established^4^,^9^,^10^,^11^. It is currently assumed that in ionic conductors, in the same way as in semiconductor p-n or Schottky junctions, the ensuing electrostatic fields are screened by mobile charges over a space charge layer of thickness λ^*^ determined by the Debye screening length *L*_*D*_ of the material, which allows an experimental estimation of *L*_*D*_ within the Mott-Schottky model^12^. However, a discrepancy is often encountered^4^,^12^ since reported *L*_*D*_ values are about one order of magnitude larger than expected from the Debye-Hückel theory^13^. Previous attempts to obtain microscopic information on ionic transport through grain boundaries made use of dielectric spectroscopy on ceramic samples^12^,^14^,^15^,^16^ but these experiments do not probe transport barriers at the atomic scale. Multiple grain boundaries with varying properties, as well as unknown ionic pathways *across* as much as *along* grain boundaries, are being averaged when measuring the electrical response of macroscopic ceramic samples. The poor knowledge of the structure and composition changes at the grain boundaries further obscures interpretation of the data.

A paradigmatic case is YSZ [*y* Y_2_O_3_: (1 − *y*) ZrO_2_], an ion conductor extensively used as electrolyte in solid oxide fuel cells (SOFC)^1^. In YSZ, doping with Y_2_O_3_ is known to stabilize the cubic fluorite structure of ZrO_2_ and to supply the oxygen vacancies responsible for the conductivity. For typical dopant concentrations (about 2.5 10^27^ m^−3^ for *y* = 9%) at intermediate temperatures (300 °C) the theoretically predicted value of the Debye screening length is only ~1 Å. At the same time the values inferred from Dielectric Spectroscopy experiments on YSZ ceramics is ~1 nm^12^. These findings cast doubt on the notion that space charge layers control grain-boundary barriers in ionic conducting materials like YSZ. The overall validity of the Debye model in YSZ has been disputed^17^,^18^,^19^,^20^,^21^,^22^,^23^.

Here we show that, contrary to the current belief, there is no oxygen vacancy depletion layer screening a positive charge build-up at YSZ grain boundaries. Instead we find that *structural* oxygen vacancies are present within ~1 nm at the grain boundary and their positive charge is compensated by negatively charged acceptor states localized at the grain boundary plane. This result is at odds with the current understanding of ion transport through grain boundaries based on charge screening by mobile ions as described in terms of space-charge models. Our conclusions are derived through analysis of the chemistry, structure, and transport of a *single* grain boundary in an YSZ bicrystal. We combine state-of-the-art electron microscopy and spectroscopy with sensitivity and resolution sufficient to probe the oxygen distribution within any space charge layer, contact microscopy with electrochemical contrast, and dielectric spectroscopy on artificially patterned microstructures, together with density functional theory (DFT) based calculations.

For our study, we used YSZ bicrystals of the utmost quality, with an interface smooth on an atomic scale. Figure 1(a) shows an atomic resolution Z-contrast image of one of our grain boundaries (GB), where a perfect array of evenly distributed dislocation cores can be observed. Since such dislocation cores in complex oxides tend to be non-stoichiometric, we acquired electron energy-loss spectrum images to study the grain boundary chemistry. Concentration (normalized integrated signal) maps corresponding to the Zr *L*_*2,3*_, Y *L*_*2,3*_, and O *K* edges are shown in panels (b–d). The chemical composition obtained from these electron energy-loss spectroscopy (EELS) maps exhibits large deviations from the bulk on the grain boundary dislocation cores, as typically observed at grain boundaries^24^,^25^,^26^,^27^,^28^. Panel (e) shows concentration changes of each atomic species as a function of the distance to the boundary plane. As hinted by previous lower resolution STEM observations^29^,^30^,^31^,^32^, there is a strong yttrium segregation, doubling the relative Y concentration at the dislocation cores. This local Y enrichment is noticeable to the eye from the bare inspection of the EEL spectra (see panel (f) where a spectrum from the GB core and bulk are compared). Elemental maps corresponding to the Zr *M*_*4,5*_, Y *M*_*4,5*_, and O *K* edges exhibit similar trends, summarized in Supplementary Fig. S4. Also, the concentration of oxygen decreases significantly close to the boundary plane, much more than expected from the measured Zr/Y stoichiometry (black line). In terms of O content, such change would be stoichiometrically equivalent to a full substitution from ZrO_2_ into Y_2_O_3_, even when the cation concentration does not change accordingly. This O defficiency can, therefore, be regarded as *structural* vacancies, introducing non-stoichiometry, as found previously in the case of grain boundaries in SrTiO_3_ and YBa_2_Cu_3_O_7_^27^,^33^. Interestingly, the length scales associated with the inhomogeneity of Y and O concentrations on the boundary (as obtained from the FWHM of the EELS compositional profiles) is in the 0.5 nm range for both species (one unit cell). We find no evidence of any region *depleted* of oxygen vacancies. By solving the Poisson equation, we have calculated the negative charge density and extension of the expected vacancy depletion region according to the space charge model, and it is clearly absent in the experimental data (see Supplementary Materials). This finding disagrees with previous understanding and currently established models for ionic transport through the grain boundary^12^,^34^,^35^,^36^,^37^,^38^, which are based on the existence of an oxygen-vacancy-depleted space-charge layer that is several nanometers thick and screens the electrostatic field created by the excess oxygen vacancies at the grain boundary core. Note, however, that the absence of space charge layers may not be a general result for all grain boundaries in ionic conductors.

In order to characterize ion transport across a single boundary we performed dielectric spectroscopy measurements using these bicrystals, and we indeed found that ion conductivity across the boundary is strongly depressed (see Supplementary Materials for a detailed analysis). Figures 2(a,b) show the complex impedance at several temperatures where the dielectric relaxations of the bulk, grain boundary and electrodes can be separated and are observed at the highest, intermediate and lowest frequencies respectively. If a space-charge region were associated with each side of the grain boundary, when the frequency of the ac electric field is low enough, positive and negative charge would alternately accumulate at each side of the grain boundary, separated by a distance 2λ^*^, due to the blocking of mobile ions. λ^*^ would be the thickness of the space charge layer at each side of the grain boundary. From the ratio between the bulk (*C*_*b*_) and grain boundary (*C*_*gb*_) capacitance values (see Fig. 2(c)), by using the expression *C*_*b*_/*C*_*gb*_ ≈ 2λ^*^/*d*_*e*_, where *d*_*e*_ is the effective distance between electrodes [see Supplementary Materials], we can estimate a value of λ^*^ ≈ 4 ± 1 Å, of the order of the size of one unit cell. This value is in excellent agreement with the STEM-EELS results for the extent of the oxygen depletion on either side of the grain boundary, pointing to an intimate connection between the structural vacancies and the observed blocking. We want to emphasize that this value is about one order of magnitude smaller than previous estimates from ceramic samples^12^. The oxygen vacancies at the grain boundary are structural, not the result of space-charge formation according to the Schottky model (see theory below). Nevertheless, a fit of the data to the Schottky model would yield △φ = 0.35 ± 0.01 V for the electrostatic barrier at 275 °C and a value λ^*^ = 4.7 ± 0.9 Å for the thickness of the space charge layer (see Supplementary Figures S1 and S2). This value of λ^*^ reflects the correct value of the extent of the oxygen vacancy concentration^39^, but this is coincidental since the underlying thesis of the Schottky model is not applicable.

Although ion transport is strongly blocked through the boundary plane, the large concentration of *structural* O vacancies at the grain boundary might give rise to an enhancement of ion conductivity within this region. In order to probe local ion conductivity close to the grain boundary plane we have conducted nanoscale mapping of electrochemical activity using electrochemical strain microscopy (ESM)^10^,^40^,^41^. For the first time, ESM has allowed obtaining direct images of ion blocking at the boundary (see Supplementary Materials for a detailed description). Figure 3(a) shows hysteresis loop opening maps, offering a measure of the ionic mobility (area under the loop) and critical potentials required to induce vacancy creation/annihilation in the tip-surface junction (inflection points). The hysteresis loops in the ESM response at the grain boundary and bulk regions have been obtained from the regions within green and red rectangles respectively (see Fig. 3(c)). It can be observed that loops at the grain boundary region are statistically significantly different than in the bulk. A smaller loop opening is indicative of a much lower mobility of oxygen ions. Figure 3(b) is a topographic map showing the presence of a step of about 1 unit cell height at the bicrystal boundary, suggesting only minimal potential for topographic cross-talk (which is further minimized through the use of band excitation detection^42^,^43^).

Figures 3(d–f) also display surface ESM scans at zero dc bias but varying ac signal levels. For small ac biases (confined within the region where hysteresis loops in Fig. 3(a) coincide), the images are featureless. At the same time, for ac levels higher than the threshold for loop opening, a clearly visible feature can be observed at the grain boundary. It is important to emphasize that topographic crosstalk can be excluded as the reason for the differences in the loop openings since crosstalk would appear irrespective of voltage and it would also give rise to an anomaly at the grain boundary in the corresponding resonant frequency map, which is not observed. ESM provides a direct image of how the grain boundary acts as a barrier for ion transport. However, because the signal generation volume in ESM is limited by tip radius and effective contact radius as well as transport lengths its spatial resolution is limited to the order of 100 nm. ESM is a surface technique and, like any other SPM probe based technique, is limited in its ability to extract three-dimensional information beyond the interaction volume of the tip with the surface. In any case, despite the limitations from ESM data to discuss the ion kinetics, the ESM images clearly indicate the blocking nature of the grain boundary (similar to e.g. studies of the GB by Kelvin probe force microscopy performed earlier^44^).

Our STEM-EELS data prove that we have Y enrichment and also O depletion within 0.5 nm of the grain boundary, but no evidence of any region *depleted* of oxygen vacancies. Hence, the observed blocking of oxygen ions cannot be explained by using the currently established (space-charge) models. We show next which the origin is of the barriers for ion transport through the grain boundary. Theoretical calculations by means of density-functional theory (DFT), as detailed in the Supplementary Material, provide an atomic-scale understanding of the nature of the transport barrier. First, we estimate the formation energy of oxygen vacancies. The results indicate that, even in the absence of Y, oxygen vacancies are favoured both in the grain boundary and also in the adjacent atomic planes, lowering the energy of the GB by more than 0.1 eV/Å^2^. This finding is consistent with results from other oxides^26^,^27^,^33^, and proves that the observed enhancement of the concentration of oxygen vacancies in STEM-EELS images is, indeed, an intrinsic phenomenon. The calculations further find that Y segregates in the grain boundary (segregation energy ~2.91 eV) and that Y segregation enhances the oxygen vacancy concentration by lowering the pertinent formation energy. This is likely due to the relative negative charge of yttrium ions. Note, however, that the positive charge resulting from the oxygen vacancies at the grain boundary is not fully compensated by the extra yttrium ions nearby.

Interestingly, two electrons accompany the creation of each oxygen vacancy. In an otherwise undoped perfect crystal, the electrons go in the conduction bands and dope the material n-type. We find, however, that a grain boundary has empty electronic states in the energy gap (see Fig. 4). Thus, the vacancy electrons are captured in these states, causing a minor charging of an otherwise pristine grain boundary (the electric field is significantly screened because of the large dielectric constant). The fact that the vacancy electrons go to states in the gap has a secondary effect besides charge redistribution: the formation energy of uncompensated oxygen vacancies is much lower than in bulk. This is because the electrons doped by the vacancy no longer need to go into localized states around the vacancy (Fig. 4(b)); they go instead to the energetically more favourable states right at the grain boundary (Fig. 4(c)). This localization actually is the origin of the space charge at the boundary. The inclusion of Y in the calculations makes it even more favourable for electrons to migrate to the grain boundary plane, since Y acts as an acceptor (as it does in bulk) and thus this picture prevails (Fig. 4(d)). Our DFT calculations indicate that charge neutrality is not achieved by an oxygen-depleted vacancy layer, but rather by a negatively charged core created by acceptor states at the grain boundary. Thus, oxygen vacancies arriving at a grain boundary initially face an attractive potential because of the mild negative charge on grain boundaries. For transport through the grain boundary to take place, vacancies would arrive on one side and depart from the other side. However, for this process to happen they would have to climb out of an electrostatic well that attracts them toward the grain boundary, naturally resulting in an increase of the barrier for ion transport. Finally we would like to mention that, as observed from the DOS calculations (see Fig. 4), the extra electrons fill the p orbitals of cations, modifying the chemical bonding at the interface. Factors like abundant dangling bonds may also have an effect on ion transport at the grain boundary.

In summary, combined theoretical calculations, ESM, STEM-EELS, and Dielectric Spectroscopy measurements in a single YSZ grain boundary were used to study the mechanisms underlying why a grain boundary acts as a barrier for ion transport. Chemical quantification through STEM/EELS shows that important chemical changes take place within the plane of the grain boundary that extends to ~1 nm. An electrostatic potential dip appears at the grain boundary caused by lower formation energy and by mild negative charging that compensates the positive O vacancies. The reduction in ionic conductivity is caused by the need for mobile vacancies to emerge from the potential dip when they cross a grain boundary. The immobile vacancies, structural in origin, significantly reduce the energy of the grain boundary (compared to a stoichiometric one), while the acceptor states, resulting from electronic reconstruction at the grain boundary, act as a charge reservoir for the electrons donated by the structural vacancies. Our findings constitute a major advance towards a reliable characterisation and understanding of ionic transport at interfaces, which will help in establishing the physics underlying the emerging field of nanoionics, as well as in optimizing the performance of solid state electrochemical devices at the nanoscale.

## Methods

YSZ bicrystals with 9% mol yttria content and a symmetrical 33° [001] tilt grain boundary were acquired from MaTeck GmbH. Bicrystals were made by means of solid phase intergrowth. Single crystals were cut to obtain surfaces with desired orientations. A pair of single crystals aligned along the oriented surfaces was annealed under pressure applied normal to the conjugated surfaces in ultra high vacuum. Usual process conditions (annealing at 1873 K for 15 h in air) prevent plastic deformation of the crystals, and provide an interface smooth on an atomic scale, free from impurities and deformation dislocations. The bicrystals were cut and polished with the surface vector in the (001) orientation. The average lattice parameter along with the chemical composition were checked by x-ray diffraction and EDX microanalysis.

STEM-EELS measurements were carried out in an aberration corrected Nion UltraSTEM200 operated at 200 kV and equipped with a Gatan Enfinium spectrometer and in a Nion UltraSTEM100 operated at 100 kV, also equipped with a 5^th^ order aberration corrector and a Gatan Enfina EEL spectrometer. This electron microscope can routinely produce a sub-Ångström electron beam with a full width at half maximum around or below the 0.08 nm range. For spectrum imaging, the electron probe is scanned along the region of interest and an EEL spectrum is acquired in every pixel. Principal component analysis was used to remove random noise. Specimens were prepared by conventional methods: grinding and ion milling. More than 50 images and simultaneous EELS spectra were taken in three different specimens, that were coated with a 1 nm thick layer of Ir to prevent charging. The STEM specimen thickness was kept in the 0.2–0.3 inelastic mean free paths (25–30 nm) range in most cases although on occasion thicker samples were used to evaluate the role of electron beam broadening.

**Figure 2:**
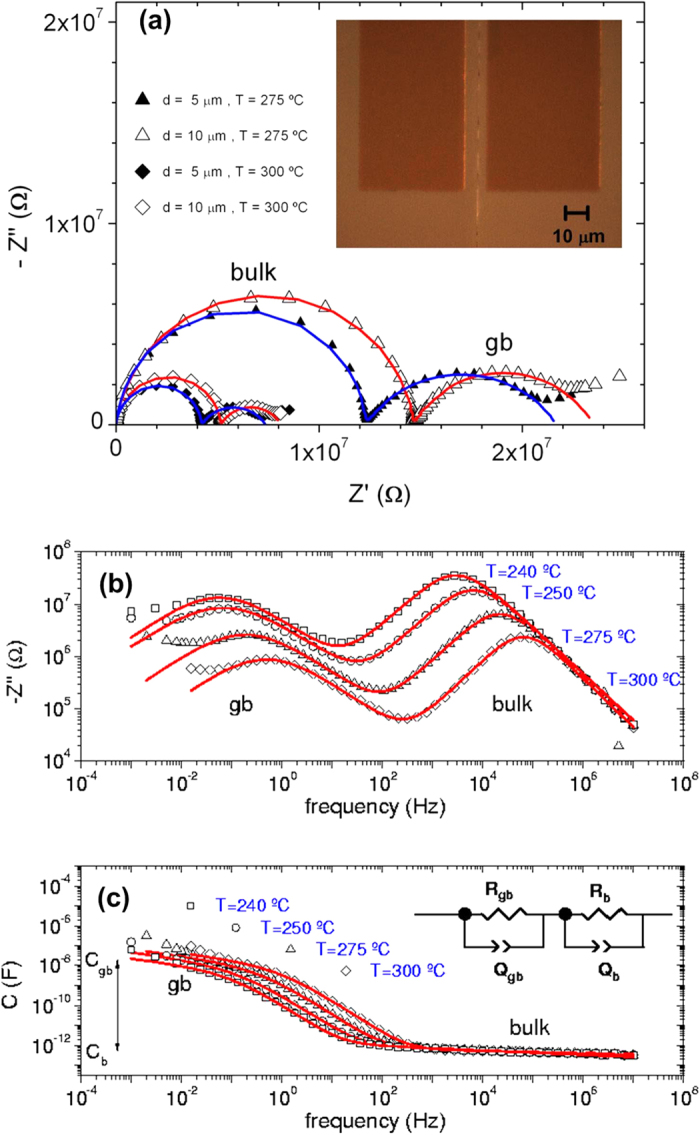
Dielectric spectroscopy of YSZ bicrystal. (**a**) Complex impedance plots at 275 °C (triangles) and 300 °C (diamonds) showing the contributions to ionic transport due to the bulk (left semicircle) and to the grain boundary (gb) (right semicircle) in YSZ bicrystals with electrodes separated *d* = 10 μm (open symbols) and 5 μm (solid symbols). Figure Inset: Optical microscopy image of the bi-crystalline boundary between the two gold electrodes. Frequency dependence of the imaginary part of the impedance (**b**) and of the capacitance (**c**) at several temperatures ((•) 240 ºC, (⌍) 250 ºC, (∆) 275 ºC, (◊) 300 ºC) for a sample with electrode separation *d* = 10 μm. The frequencies *ω*_*gb*_ and *ω*_*b*_ (as referred in the text) are the peak frequencies observed at each temperature in the *Z*” spectra for the grain boundary (low frequency) and bulk (high frequency) contributions. Solid lines are fits to the equivalent circuit shown in the sketch (see Supplementary Materials).

Electron beam lithography and sputtering techniques were used to pattern and deposit gold electrodes (2 mm long and 50 μm wide) on the sample surface, separated by *d* = 5 and 10 μm with the boundary in between (see inset to Fig. 2). The micron-size electrode distance was necessary to render the grain boundary resistance *R*_*gb*_ comparable to that of the bulk resistance *R*_*b*_. Dielectric spectroscopy measurements in the frequency range 10^−3^–10^7^ Hz and at temperatures between 240–300 °C were conducted using a Novocontrol BDS-80 system. A small amplitude of the ac voltage *V*_*ac*_ = 50 mV was used to assure linear response of the system.

Atomic force microscopy (AFM) and ESM measurements were performed with a commercial system (Asylum Research Cypher) additionally equipped with LabView/MatLab based band excitation controller implemented on a NI-5122/5412 fast AWG and DAQ cards. ESM imaging and spectroscopy was performed with 200–400 kHz 2 V_pp_ band excitation signal applied to a metal coated tip. The spectroscopic measurements were performed at ~1s/pixel waveform with 2 ms at each dc voltage step. Mapping of the electromechanical response was done typically on a 50 × 50 points grid with a spacing of 20 nm, albeit other spacing and image sizes were also used.

The theoretical energies and the atomic and electronic structures of the bicrystal were obtained within density functional calculations, in particular within the generalized gradient approximation developed by of Perdew, Burke and Ernzerhof. Projector Augmented-Wave Method (PAW) as implemented in the VASP code was utilized. The planewave cutoff for all calculations is 400 eV, and energies were estimated with gamma point calculations.

## Additional Information

**How to cite this article**: Frechero, M. A. *et al.* Paving the way to nanoionics: atomic origin of barriers for ionic transport through interfaces. *Sci. Rep.* **5**, 17229; doi: 10.1038/srep17229 (2015).

## Supplementary Material

Supplementary Materials

## Figures and Tables

**Figure 1:**
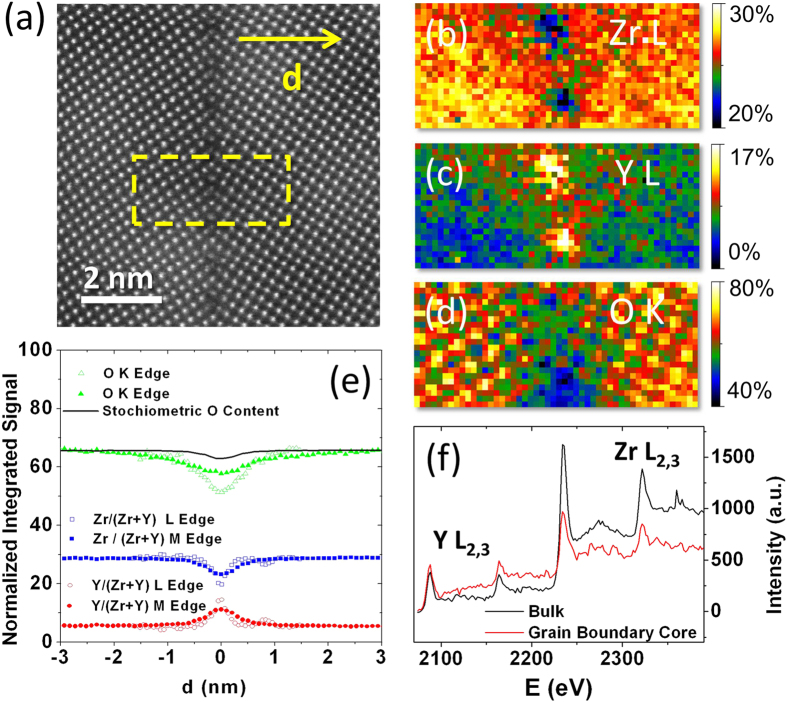
STEM-EELS of YSZ bicrystal. Z-contrast image of the grain boundary region obtained in a Nion UltraSTEM 200 operated at 200 kV (**a**), the yellow dashed box marks the area where an EEL spectrum image was acquired. (**b**–**d**): Atomic resolution, integrated signal maps of Zr *L*_*2,3*_, Y *L*_*2,3*_ and O *K* edges, respectively, normalized to the nominal bulk concentration. The exposure time is 0.1 s per pixel (**e**) Normalized integrated signal profiles across the direction marked with an arrow on (**a**). Open symbols correspond to the quantification based on the analysis of the O *K* and Zr and Y *L*_*2,3*_ edges in (**d**). Solid symbols result from a quantification performed on a spectrum image including the O *K* and the Zr and Y *M* edges instead (see Supplementary Information). Zr and Y profiles have been normalized to the total cation concentration. The black line is the stoichiometric O content that would be expected from the measured Zr and Y signals alone. Error bars (noise) are of the order of 1–2%. (**f**) Averaged Y *L*_*2,3*_ and Zr *L*_*2,3*_ EEL spectra from the bulk crystal (black) and the dislocation core (red).

**Figure 3:**
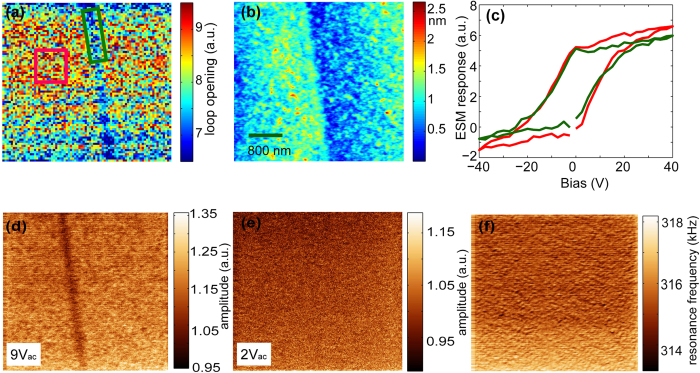
Electrochemical strain microscopy of YSZ bicrystal. (**a**) ESM loop opening map across the grain boundary (**b**) topography observed across the boundary (**c**) selected ESM loops observed on and adjacent to the grain boundary. The green and red loops are selected from the marked regions in (**a**). (**d**) The grain boundary is clearly observed in line mapping with 9 V ac. (**e**) Line mode mapping with 2 V ac (**f**) minimal frequency change is observed across the boundary which rules out topographic cross talk.

**Figure 4:**
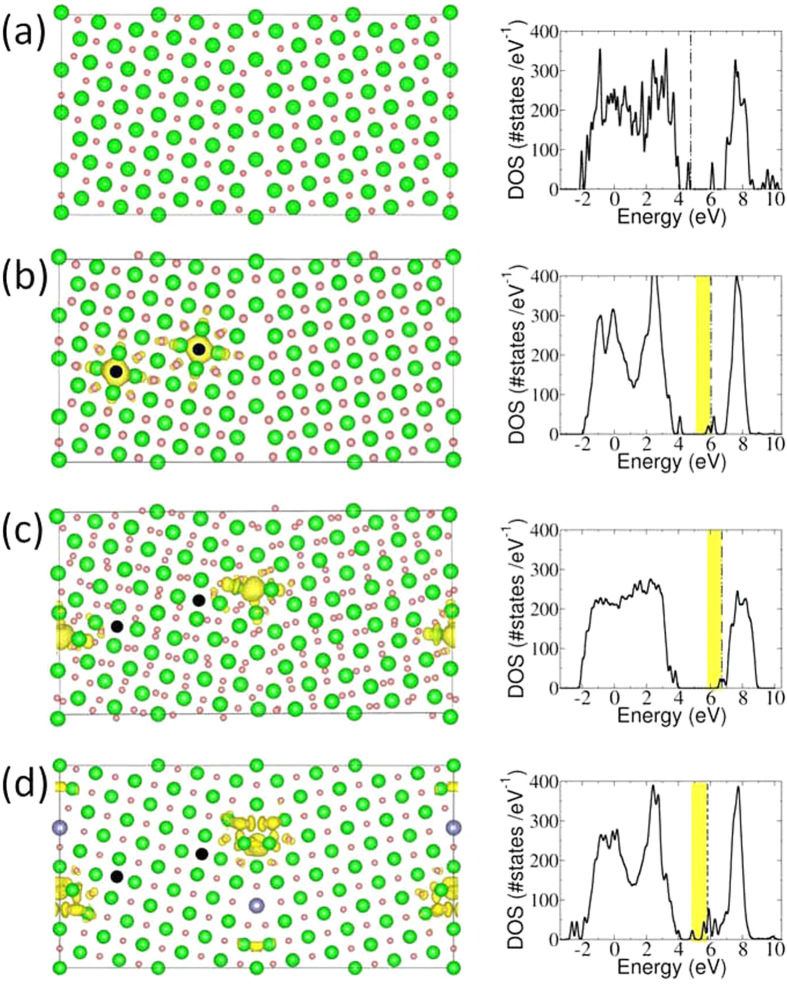
Charge distribution according to DFT calculations. (**a**) Projection of the stoichiometric grain boundary along with the density of states (DOS). Zr, and O atoms are represented by light green and pink spheres. A complementary grain boundary lies at the cell edge in order to obtain a periodic unit cell. The vertical line in the DOS plot marks the position of the Fermi energy. (**b**) Projection and DOS for a grain boundary with oxygen vacancies (in black), before structural optimization. A symmetric configuration is chosen to avoid spurious effects due to the periodicity of the system. The charge density (in yellow) over the atomic structure corresponds to the electrons doped by the oxygen vacancies (also marked in the DOS), they are localized around the vacancies, making then neutral. (**c**) Atomic structure, DOS and charge distribution of the same O vacancies as in (**b**), after structural relaxation. The charge resulting from the vacancies goes now to the grain boundary, forming a space charge layer consistent with microscopy and transport measurements. (**d**) Same as (**b**) but with Y (in purple) enrichment in the crystallographic position consistent with the microscopy images. Upon Y doping, localization of the charge at the grain boundary is more favourable, even in the absence of structural relaxation.
