# Supplementary material for: Paving the way to nanoionics: atomic origin of barriers for ionic transport through interfaces
Source: Sci Rep. 2015 Dec 17;5:17229. doi: 10.1038/srep17229 (PMC4682188; doi:10.1038/srep17229)
Supplement: Supplementary Materials [file srep17229-s1.pdf]

Supplementary Information

Paving the way to nanoionics: atomic origin of barriers for ionic transport through interfaces

M. A. Frechero[1,†] , M. Rocci[1], G. Sánchez-Santolino[1,2], Amit Kumar[3], J. Salafranca[1,2], Rainer Schmidt[1], M. R. Díaz-Guillén[1], O. J. Durá[1], A. Rivera-Calzada[1], R. Mishra[4,2], Stephen Jesse[3], S. T. Pantelides[2,4], Sergei V. Kalinin[3], M. Varela[2,1], S. J. Pennycook[2], J. Santamaria[1] and C. Leon[1,*]

[1] *GFMC, Departamento de Física Aplicada III, Facultad de Física, Universidad Complutense de Madrid, Campus Moncloa, 28040 Madrid, Spain*

[2] *Materials Science & Technology Division, Oak Ridge, TN 37831, USA.*

[3] *Center for Nanophase Materials Sciences, Oak Ridge, TN 37831, USA.*

[4] *Department of Physics and Astronomy, Vanderbilt University, Nashville, TN 37235, USA*

[†] *On leave from Departamento de Química-INQUISUR, Universidad Nacional del Sur, Av. Alem 1253, 8000 Bahía Blanca, Argentina.*

Broadband Dielectric Spectroscopy.

Separation of bulk and grain boundary contributions to dielectric response

In order to obtain the dc bulk (R_b) and grain boundary (R_{gb}) resistances at each temperature, we fit the impedance data to an equivalent circuit where bulk and grain boundary contributions to the total impedance Z^* add in series:

$$Z^* = Z_b^* + Z_{gb}^* \quad . \qquad \text{(S1)}$$

Each separate relaxation (i = b for bulk, and i = gb for grain boundary) has been modelled using a parallel resistor-capacitor combination [s1]:

$$\frac{1}{Z_i^*} = \frac{1}{R_i} + Q_i\,(j\omega)^{n_i} \, . \qquad \text{(S2)}$$

In this expression the ideal capacitor has been replaced by a constant phase element (CPE) Q_i in order to account for the universal Jonscher's response that is commonly observed in ionic conductors [s2-s4]. The results from the fits to Eqs. (S1) and (S2) are shown as solid lines in Figure 2. Note that the data at the lowest frequencies have been excluded from the fits, because they are affected by electrode blocking effects which are not accounted for in the equivalent circuit model.

Determination of the built-in electrostatic potential and space charge layer thickness at the boundary.

According to the Schottky model, the value of the built-in potential $\Delta\Phi$ at the boundary plane can be determined from Dielectric Spectroscopy measurements by using the expression

$$\frac{\rho_{gb}}{\rho_b} \approx \frac{\omega_b}{\omega_{gb}} \approx \frac{\exp(2e\Delta\Phi / kT)}{4e\Delta\Phi / kT} \quad , \qquad \text{(S3)}$$

from the grain boundary (ρ_{gb}) and bulk resistivity (ρ_b) values obtained at each temperature, or alternatively from the frequencies at which the grain boundary and bulk semicircles, ω_{gb} and ω_b respectively, present their maxima [s5]. These frequencies are easily determined from Figure 2(b). Note that by measuring a single grain boundary in a bicrystal, the characteristic relaxation frequencies for the grain boundary and bulk conduction processes can be directly obtained from experimental data without simplifying assumptions, unlike in previous works dealing with ceramic samples [s5, s6]. We have thus analyzed our Dielectric Spectroscopy measurements to check the validity of the Schottky model to explain the results obtained just in terms of an electrostatic potential barrier. $\Delta\Phi$ values calculated from Eq.(S3) are plotted in Fig. S1

as a function of temperature. We find that the built-in potential is ΔΦ = 0.35 ± 0.01 V at 275 ºC, which is larger than previously reported values from ceramic samples (ΔΦ ≈ 0.20 - 0.25 V at the same temperature) [s5, s6]. Furthermore, we find a temperature dependence of ΔΦ which could be explained as follows: Both the grain boundary and bulk resistivity are found to be thermally activated (see inset to Fig.S1), showing essentially the same activation energies within experimental error (E_b = 1.16±0.05 eV and E_{gb} = 1.17±0.04 eV) and in good agreement to previous results in single crystal and ceramic YSZ samples [s7, s8].

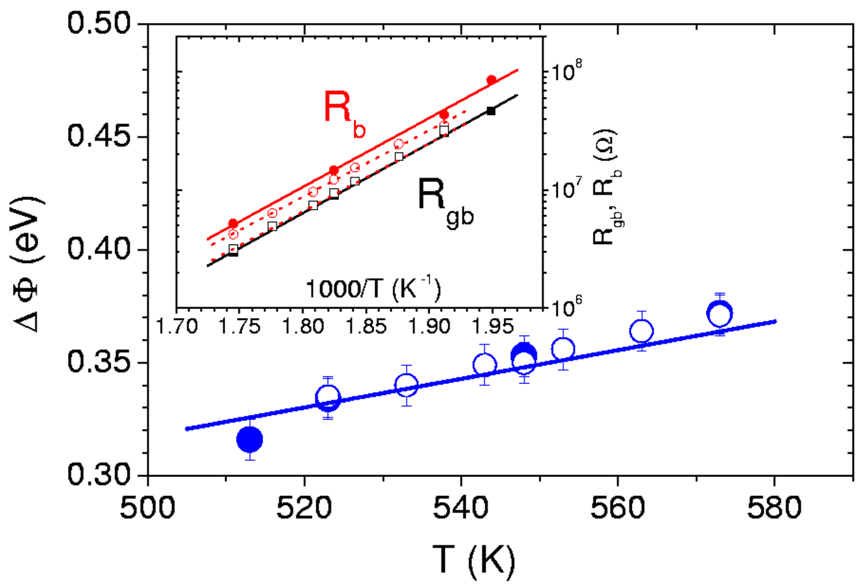

Fig. S1. Temperature dependence of the built-in potential ΔΦ = Φ(0) - Φ(∞) at the boundary or interfacial plane (x = 0) referred to its value at the bulk (x = ∞). The solid line represents the temperature dependence obtained for ΔΦ by using Eq.(S4). Inset: Arrhenius plot for the bulk (R_b) and the grain boundary (R_{gb}) resistance values. Linear fits yield activation energies E_b = 1.16 ± 0.05 eV and E_{gb} = 1.17 ± 0.04 eV. Data correspond to YSZ bicrystals with electrodes separated d=10 μm (solid symbols) and 5 μm (open symbols).

Taking the temperature derivative of Eq.(S3) leads to

$$E_{gb} - E_b = \left(2\Delta\Phi - \frac{kT}{e}\right)\left(1 - \frac{d\ln(\Delta\Phi)}{d\ln T}\right), \qquad \text{(S4)}$$

and since the activation energies E_b and E_{gb} are essentially the same, the temperature dependence of $\Delta\Phi$ must be roughly linear. Close agreement between the data and the expected temperature dependence derived from Eq.(S4) can be noticed in Fig. S1.

Figure S2 shows the space charge layer thickness λ^* as a function of temperature, as obtained from

$$\lambda^* = \left(\frac{2\varepsilon\varepsilon_0\Delta\Phi}{zen_\infty}\right)^{1/2}, \qquad \text{(S5)}$$

according to the Schottky model, with ε = 30 for the dielectric permittivity (as determined from independent measurements with a parallel plate geometry), n_∞ = 4.8 nm^{-3} for the nominal yttrium ion concentration and z = 1 for the effective charge of yttrium ions in the zirconia lattice. A value of λ^* = 4.7±0.9 Å is obtained at T = 275 ºC and, as shown in Fig. S2, the values obtained for λ^* show a slight increase with increasing temperature. The Debye length L_D can be determined according to the Debye-Hückel theory [s9] from the dopant concentration n_∞ and the dielectric permittivity ε of the material as:

$$L_D = \left(\frac{\varepsilon\varepsilon_0 kT}{2z^2e^2n_\infty}\right)^{1/2}, \qquad \text{(S6)}$$

where k is Boltzmann's constant, T is temperature and z the charge in electron units. By using Eqs (S5) and (S6) the following relation is obtained,

$$L_D = \lambda^*\left(\frac{kT}{4ze\Delta\Phi}\right)^{1/2}, \qquad \text{(S7)}$$

and therefore the Debye length can also be estimated from the values of λ^* and determined from experimental data (see Figure S2).

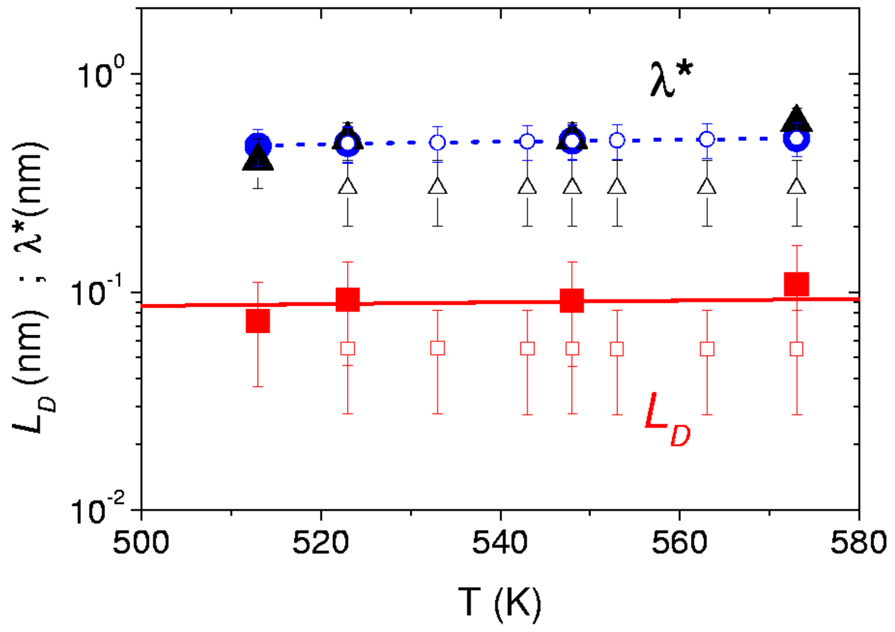

Fig. S2. Space charge layer thickness (λ*) determined from Eq. (S5) for a Schottky barrier model (circles, dotted line) and determined directly from the ratio between grain boundary and bulk capacitances (triangles). Squares represent the Debye screening length determined in the present work for YSZ at several temperatures, and the solid line represents its theoretical value and temperature dependence according to its definition (Eq.(S6)). Different data sets correspond to YSZ bicrystals with electrodes separated d=10 μm (solid symbols) and 5 μm (open symbols).

An independent estimate of the value of $\lambda^* \approx 4\pm1$ Å can be obtained from the ratio between the bulk (C_b) and grain boundary (C_{gb}) capacitance values (see Figure S2), since $C_b/C_{gb} \approx 2\lambda^*/d_e$, with the effective thickness d_e = 41.7 and 35.5 μm for d = 10 μm and 5 μm respectively (see next section).

However, we point out that these values for λ^*, of the order of just one unit cell, are too short to be consistent with the description of the space charge layer in terms of

accumulation of mobile oxygen ions to screen the electric field arising from the difference in the electrochemical potential between the bulk and the boundary plane. Consequently our results cast doubts on the validity of the Schottky model to describe the barrier for ion transport in grain boundaries and on our preliminary interpretation of the impedance spectroscopy data [s10]. We want to emphasize that the values previously inferred for the Debye screening length from Impedance Spectroscopy experiments on YSZ ceramics is ~1 nm, that is about one order of magnitude longer than the theoretical value.

In order to test the validity of a space-charge model to describe the electrostatics at the grain boundary, we have calculated the extent of a hypothetical oxygen vacancies depletion layer by solving numerically the Poisson equation. We have used a MatLab software routine to linearize and solve the corresponding nonlinear second-order ordinary differential equation, by means of the secant method. We estimate the concentration of oxygen vacancies by using the charge due to the Zr (4+), Y (3+) and O (2-) from the corresponding profiles measured by EELS. We thus calculate the profile of excess of vacancies at the grain boundary, which shows a Lorenztian shape (see Figure S3), and use it as an additional (positive) charge besides the positive charge from the cations, and the negative charge from the mobile oxygen vacancies, whose distribution has to be determined. In order to solve the Poisson equation, we assume a space-charge model where the positive charge at the grain boundary core (due to excess of oxygen vacancies, as experimentally observed from STEM-EELS) is compensated by a negative charge in an adjacent layer depleted of oxygen vacancies, which corresponds to an electric field that equals zero as boundary condition. We obtain that the lower value of the Debye length that makes the solution possible is of about 6 Å, much longer that the theoretical value (see Figure S3). Moreover, the calculated density profile of

oxygen vacancies is not consistent with the experimental EELS data, which do not show any depletion of oxygen vacancies.

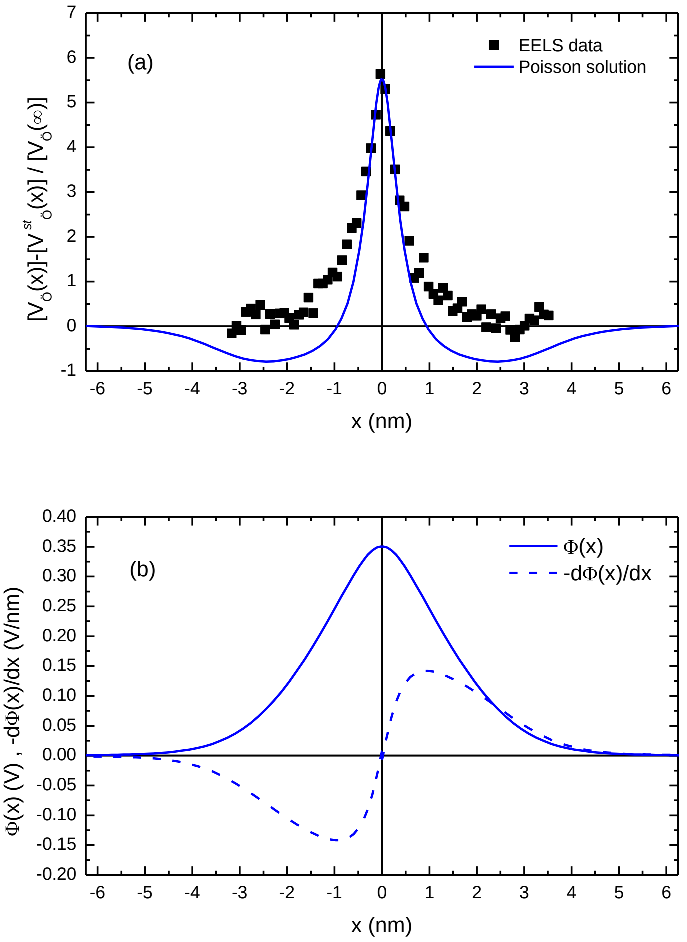

Figure S3. (a). Solid symbols correspond to the excess of oxygen vacancies, respect to the expected stoichiometric content, normalized to the bulk concentration, as calculated from EELS data. The blue line is the expected concentration of oxygen vacancies, from the numerical solution of the Poisson equation, by assuming a space-charge model (see text). The negative values would correspond to the hypothetical oxygen vacancies depletion layer, not observed experimentally by EELS. (b) Electrostatic potential (solid line) and electric field (dashed line) as a function of the distance from the grain boundary core, as obtained from the solution of the Poisson equation. We have assumed that the net charge is zero and the potential barrier is 0.35 eV, as estimated from the electrical conductivity data.

Coplanar electrodes configuration

The electrode configuration used in our experiment leads to an inhomogeneous electric field in the YSZ bicrystal as depicted in Figure S4.

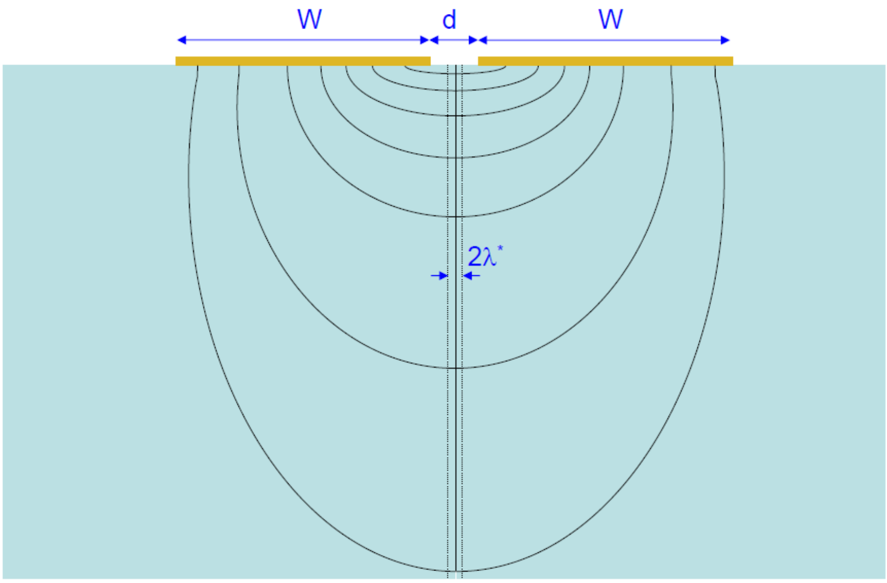

Figure S4. Sketch of the electrode geometry used and the inhomogeneous electric field created in the YSZ bicrystal. Solid lines represent electric field lines which are approximately perpendicular to the grain boundary plane within the space charge layers.

For two coplanar plates aligned parallel, of width *W* and separated by a distance *d*, as used in our experiment, the capacitance per unit length is given by [s11]

$$C_b = \varepsilon_0 \frac{(\varepsilon_r + 1)}{2} \frac{K(k')}{K(k)} \approx \varepsilon_0 \varepsilon_r \frac{K(k')}{2K(k)}$$

where $k = d/(2W+d)$, $k' = (1-k^2)^{1/2}$, and K represents the complete elliptic integral of the first kind. In the expression above it is assumed that the YSZ substrate has an infinite thickness, which is reasonable since it is significantly larger (1 mm) than the values of W (50 μm) and d (5-10 μm) in our experiment. Note that the factor $(\varepsilon_r+1)/2$ accounts for the contributions to the capacitance of YSZ (ε_r) and air ($\varepsilon_r^{air} \approx 1$), from the volume below and above the electrodes respectively. The contribution from the air volume

(above the electrodes) was neglected since ε_r (air) << ε_r (YSZ). In particular, for W=50 μm and d=10 μm, the geometric factor is $\frac{K(k')}{2K(k)} = 1.20$, while for W=50 μm and d=5 μm it is $\frac{K(k')}{2K(k)} = 1.41$. We can calculate the value of an effective distance d_e by comparing the capacitance of an equivalent parallel plate capacitor of width W and thickness d_e, $C_b = \varepsilon_0 \varepsilon_r \frac{W}{d_e}$, to the capacitance for the two parallel coplanar plate capacitor used in the actual experiment, and the values thus obtained are d_e = 41.7 and 35.5 μm for d = 10 μm and 5 μm respectively.

On the other hand, since the thickness of the space charge region at the grain boundary, $2\lambda^*$, is much lower (about four orders of magnitude) than the distance d separating the electrodes, the electric field lines within the space charge layers can be considered as perpendicular to the grain boundary (see Figure S4). This means that the planes parallel to the grain boundary and at a distance λ^* at both sides of the boundary plane, can be considered as equipotential planes, as is in fact the boundary plane itself. This approximation allows us to calculate the geometric factor for the grain boundary capacitance C_{gb} by taking into account that (1.) the capacitance of two coplanar plates aligned parallel and separated by a distance d is half the capacitance between each one of these plates and a perpendicular infinite plate situated in the middle at a distance d/2 from each; and (2.) the capacitance of two coplanar plates aligned parallel and separated by a distance d is the same as the capacitance of two similar coplanar plates but separated by a distance d-$2\lambda^*$, in series with the grain boundary capacitance C_{gb} due to the space charge region of thickness $2\lambda^*$ (see Figure S4). One can thus obtain:

$$C_{gb}{}^{-1} \approx \frac{2K(k)}{\varepsilon_0 \varepsilon_r K(k')} - \frac{2K(k_-)}{\varepsilon_0 \varepsilon_r K(k'_-)}$$

where $k = d/(2W+d)$, $k' = (1-k^2)^{1/2}$, and $k\_ = (d-2\lambda^*)/(2W+ d-2\lambda^*)$, $k'\_ = (1-k\_^2)^{1/2}$.

Substituting for the values of W and d used in our experiment it is found that:

$$C_{gb} = \varepsilon_0 \varepsilon_r \left(\frac{1}{1.01}\right)\frac{W}{2\lambda^*}$$ for W=50 μm and d=10 μm , and

$$C_{gb} = \varepsilon_0 \varepsilon_r \left(\frac{1}{1.52}\right)\frac{W}{2\lambda^*}$$ for W=50 μm and d=5 μm.

These expressions allow estimating the value of the thickness of the space charge layer, λ^*, from the ratio C_b/C_{gb} of the corresponding experimental values for the bulk and grain boundary capacitances, which can be expressed as:

$$C_b / C_{gb} = \left(1.01\ \frac{2\lambda^*}{W}\right)\left(\frac{K(k')}{2K(k)}\right) = 1.01\ \frac{2\lambda^*}{d_e}$$ for W=50 μm and d=10 μm , and

$$C_b / C_{gb} = \left(1.52\ \frac{2\lambda^*}{W}\right)\left(\frac{K(k')}{2K(k)}\right) = 1.52\frac{2\lambda^*}{d_e}$$ for W=50 μm and d=5 μm.

Finally we want to remark that since two different geometries give rise to consistent results for the microscopic parameters obtained, the experimental data provide strong support for the validity of the assumptions we have made in order to obtain the expressions above for the bulk and grain boundary capacitances, namely: (1.) 1 mm thick YSZ substrates may be considered as of infinite thickness since it is significantly larger than the width W of the electrodes used and the distance d; (2.) the electric field lines within the space charge layers are approximately perpendicular to the grain boundary plane since the space charge layer thickness λ^* is significantly shorter than the distance d between the electrodes, and (3.) the dielectric permittivity in the grain boundary is similar to its value in the bulk and the total grain boundary thickness

is two times the thickness of each (symmetric) space charge layer at both sides of the interface [s12].

Electron energy loss spectroscopy (EELS) in the STEM.

Concentration (normalized integrated signal) maps corresponding to the Zr $M_{4,5}$, Y $M_{4,5}$, and O K edges are shown in Figure S5. The EEL spectrum image was taken in the area marked as a yellow box in Figure S5(a).

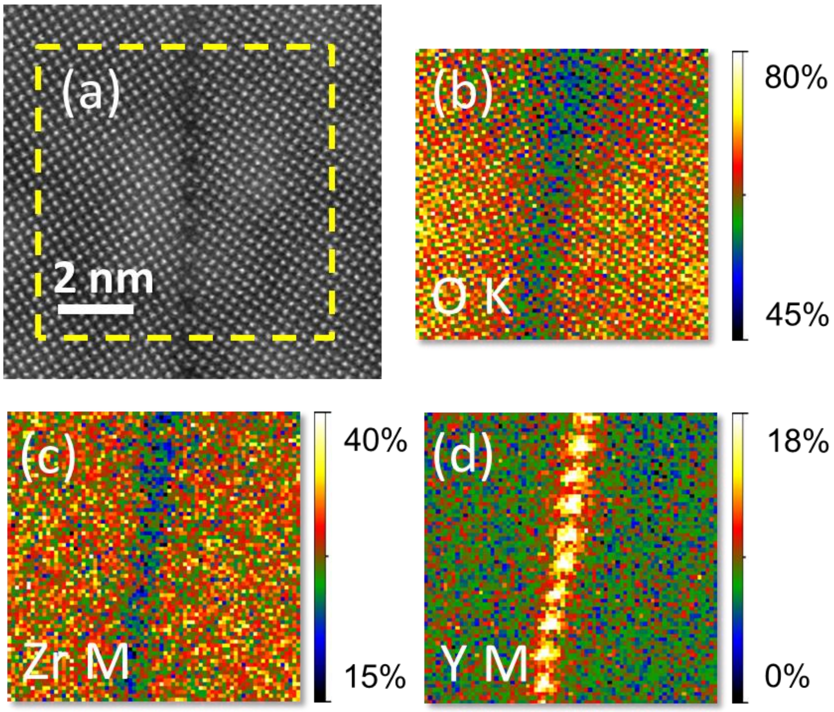

Figure S5. Z-contrast image of the grain boundary region obtained in a Nion UltraSTEM 100 operated at 100kV (a), the yellow dashed box marks the area where an EEL spectrum image was acquired. (b), (c) and (d): Atomic resolution, integrated signal maps of the O K, Zr $M_{4,5}$ and Y $M_{4,5}$ edges, respectively, normalized to the nominal bulk concentration. The exposure time is 0.05 s per pixel. Some spatial drift is observed.

Although the M- edges occur at relatively low energy-loss and are less localized than L edges, their analysis yields similar results regarding the elemental distribution. The chemical composition obtained from these EELS maps shows large changes with the

periodicity of the grain boundary dislocation cores, as also found from the analysis of Zr $L_{2,3}$ and Y $L_{2,3}$, edges (see Figure 1 in the manuscript). A strong yttrium segregation into the dislocation cores at the grain boundary is observed. The concentration of oxygen vacancies increases close to the boundary plane. The concentration changes of each atomic species as a function of the distance to the boundary plane are represented as solid symbols in the manuscript Figure 1(e).

It is well known that when a sample is oriented down a well defined zone axis, the EELS quantification can be affected by channeling (dechanneling). For example, the heavier columns may be enhanced in a thin crystal (as the beam is focused onto the column). Conversely, their relative signal may be reduced in thicker specimens as the beam is scattered away from the column. In order to examine the role of channeling, we have examined a number of different sample thicknesses and orientations. For example, Figure S6 shows an EEL spectrum image that has been acquired with the left side of the bicrystal aligned on- axis while the right side is off-axis. The quantification results plotted in Fig. S6(b) show a comparison between the perfectly on-axis quantification from Figure 1 in the manuscript (blue curve) and the SI acquired in the region marked with a yellow box in Fig. S6(a) (red curve). The profiles show how for the left side of the grain boundary, where both SI have been acquired in a zone axis orientation, the values for the oxygen concentration are extremely similar, as expected. On the other hand, the analysis on the right, off-axis side yields a slight difference of around 1% larger O relative signal when compared with the on-axis result. It is worth noting that such a minor difference is near the error bars for the quantification method, so it might not be relevant. Nevertheless, the increased oxygen relative content measured in the off-axis side of the GB suggests that the signal from the heavier Zr/Y columns may be

slightly enhanced in the on-axis channeling conditions for this specimen thickness (0.5 inelastic mfp). In summary, while these profiles prove how channeling may affect the EELS quantitative analysis, the effect is relatively minor: around one order of magnitude smaller than the changes in the oxygen concentration that we found in the grain boundary region. Hence the basic conclusions of the work are not altered by any effect due to channeling.

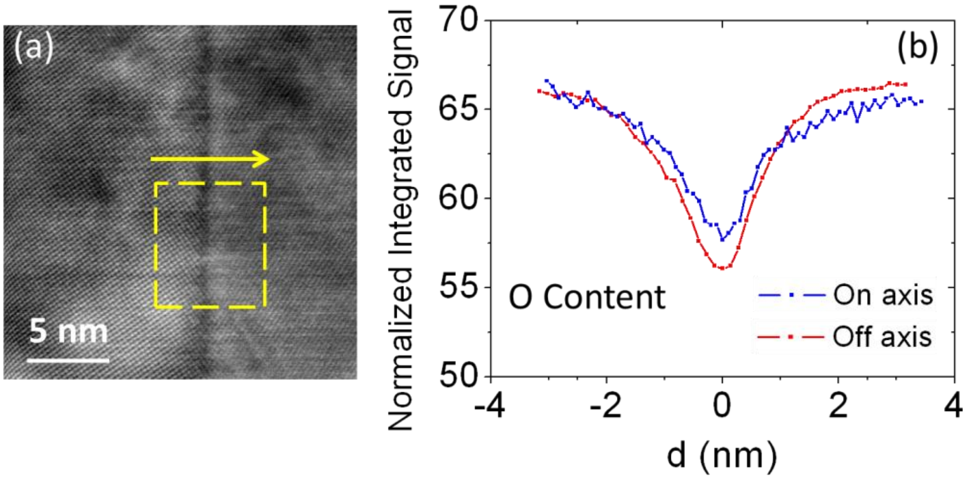

Figure S6. Dependence of the STEM-EELS quantification on the tilt angle. (a) Z-contrast image of the YSZ grain boundary (GB) region where the left side of the GB plane is tilted on zone axis and the right side is off-axis. The image was obtained in a Nion UltraSTEM 200 operated at 200kV, the yellow dashed box marks the area where an EEL spectrum image was acquired. (b) Normalized integrated signal profiles across the direction marked with an arrow on (a), averaged along the direction parallel to the GB plane. The blue curve shows the oxygen profile from the on-axis SI represented in Figure 1 of the manuscript, while the red profile shows the oxygen profile obtained from the SI from the region marked in (a).

Electrochemical Strain Microscopy.

In Electrochemical Strain Microscopy (ESM), a biased SPM probe concentrates the electric field in a small volume of material, resulting in ion motion. Associated change in the molar volume results in the vertical surface displacement, which can be detected by the AFM detection system with the 2-4 pm resolution. The response is then related to the local ionic mobility and electrochemical reactivity.

For high frequencies, the ESM signal (dominated by diffusion processes since migration effects are insignificant [s13-s15]) was analyzed in the diffusion approximation by Morozovska et al. and was shown to be

$$u_3(0,\omega) \approx (1+\nu)\beta C_S\sqrt{\frac{2D}{\omega}} ,$$

where ν is Poisson's ratio (typically 1/3), β is the Vegard expansion coefficient, ω is frequency, and C_s is tip-induced concentration change. The latter is related to voltage as $C_s = \left(\frac{\partial U_S}{\partial C}\right)^{-1} V_0$, where $\partial U_S/\partial C$ is the slope of charge-voltage curve. From this formula, we estimate that the ESM signal is directly proportional to diffusion coefficient. From the data in Fig 3, the reduction of contrast by ~15% over 100 nm length will be consistent with complete blocking of ionic motion over 4 nm length, i.e. close to the STEM prediction.

Density Functional Calculations.

As mentioned in the methods section, the calculations utilized the paramatrization of the exchange-correlation functional by Perdew, Burke and Ernzerhof [s16]. Projector Augmented-Wave Method (PAW) [s17] as implemented in the VASP code [s18] was utilized, with planewave cutoff of 400 eV, and energies estimated with gamma point-only calculations.

Our starting point is an ideal (twinned) bicrystal with two symmetric grain boundaries (GBs), obtained by a “cut and turn” procedure on a ZrO_2 supercell with cubic fluorite structure. We choose a lattice parameter of ~5.13 Å that corresponds to the minimum energy for cubic ZrO_2 within the present approximations. For a proper matching at both GBs the supercell dimensions then need to be 37.01Å, 18.50Å and 5.13Å (or integer multiples of them). Within this procedure, the coincidence site lattice still has a few atomic columns symmetrically placed very close to the GB that are unphysically close to each other. After removal of the atomic columns inconsistent with the microscopy images, the chemical formula for the supercell was $Zr_{100}O_{200}$ (all atomic columns present in the microscopy images are assumed to have full occupancy). We performed structural relaxations with a converge criterion of forces < 0.01 eV/Å . After relaxation, the lattice parameters in the directions parallel to the GBs remain very similar (difference < 0.4%). The direction perpendicular to the GB changes with relaxation and depends on the number of vacancies. Due to the computationally intensive calculations we choose to keep the unit cell fixed to the stoichiometric composition when determining energies for different oxygen contents. This is appropriate since further cell relaxation can only lower the formation energy of oxygen vacancies.

The “unrelaxed” structures in Fig. 4 were obtained by direct removal of symmetrically placed oxygen atoms from the stoichiometric cell. In this way, the spurious effects resulting from the periodicity in the potential imposed by the boundary conditions is minimized. When indicated, relaxation is carried out until forces are less than 0.01eV/Å. For the Y doped grain boundary the Y atoms were substituted for Zr as indicated in Fig. 4, consistent with the crystallographic substitutional position inferred from the ADF images in Fig. 1.

The charge density is represented by an isosurface of the square of the modulus of the occupied wave function with energies marked by the yellow shaded area in the left panels of Fig. 4. The value of the isosurfaces displayed was adjusted for clarity (Fig 4b: 0.003 e/$Å^3$, Fig 4c: 0.0006 e/$Å^3$, Fig 4d: 0.0006 e/$Å^3$).

[s1] J. R. Macdonald, Impedance Spectroscopy. Theory, Experiments and Aplications 2nd ed. (Wiley-Interscience, 2005).

[s2] A. K. Jonscher, Dielectric Relaxation in Solids (Chelsea Dielectrics, London, 1983).

[s3] A. Rivera, J. Santamaría, C. Leon, Electrical conductivity relaxation in thin-film yttria-stabilized zirconia, Appl. Phys. Lett. **78**, 610-612 (2001).

[s4] O. J. Durá, M. A. López de la Torre, L. Vázquez, J. Chaboy, R. Boada, A. Rivera-Calzada, J. Santamaria, C. Leon, Ionic conductivity of nanocrystalline yttria-stabilized zirconia: Grain boundary and size effects , Phys. Rev. B **81**, 184301 (2010).

[s5] X. Guo and R. Waser, Electrical properties of the grain boundaries of oxygen ion conductors: Acceptor-doped zirconia and ceria, Prog. Mat. Sci. **51**, 151 (2006).

[s6] X. Guo, W. Sigle, J. Fleig, J. Maier, Role of space charge in the grain boundary blocking effect in doped zirconia, Solid State Ion. **154– 155**, 555 (2002).

[s7] A. Rivera, J. Santamaría, C. Leon, Electrical conductivity relaxation in thin-film yttria-stabilized zirconia, Appl. Phys. Lett. **78**, 610-612 (2001).

[s8] O. J. Durá, M. A. López de la Torre, L. Vázquez, J. Chaboy, R. Boada, A. Rivera-Calzada, J. Santamaria, C. Leon, Ionic conductivity of nanocrystalline yttria-stabilized zirconia: Grain boundary and size effects, Phys. Rev. B **81**, 184301 (2010).

[s9] P. Debye and E. Hückel, Zur Theorie der Elektrolyte. I. Gefrierpunktserniedrigung und verwandte Erscheinungen, Physik. Z. **24**, 185-206 (1923).

[s10] M.A. Frechero, M. Rocci, R. Schmidt, M. R. Diaz-Guillen, O. J. Dura A. Rivera-Calzada, J. Santamaria, C. Leon, "Caracterización eléctrica de fronteras de grano en conductores iónicos mediante medidas de espectroscopia de impedancias en un bicristal", Bol. Soc. Esp. Ceram. V. 51, 7 (2012).

[s11] K. J. Binns, P. J. Lawrenson, C. W. Trowbridge, The Analytical and Numerical Solution of Electric and Magnetic Fields. (John Wiley & Sons, 1992).

[s12] S. Ramanathan, J. Vac. Sci. Technol. A, **27**, 1126 (2009).

[s13] A. N. Morozovska, E. A. Eliseev and S. V. Kalinin, Electrochemical strain microscopy with blocking electrodes: The role of electromigration and diffusion, Journal of Applied Physics **111** 014114 (2012).

[s14] A. N. Morozovska, E. A. Eliseev, S. L. Bravina, F. Ciucci, G. S. Svechnikov, L. Q. Chen and S. V. Kalinin, Frequency dependent dynamical electromechanical response of mixed ionic-electronic conductors, Journal of Applied Physics **111** 014107 (2012).

[s15] L. Faulkner and A. Bard, *Electrochemical Methods. Fundamentals and Applications*, John Wiley and Sons, Inc. (2011).

[s16] J. P. Perdew, K. Burke, and M. Ernzerhof, *Generalized gradient approximation made simple*, Phys. Rev. Lett. **77**, 3865 (1996).

[s17] P. E. Blöchl, Projector augmented-wave method, Phys. Rev. B 50, 17953 (1994); G. Kresse and D. Joubert, From ultrasoft pseudopotentials to the projector augmented-wave method, Phys. Rev. B 59, 1758 (1999).

[s18] G. Kresse and J. Hafner, Ab initio molecular dynamics for liquid metals, Phys. Rev. B 47, 558 (1993). G. Kresse and J. Furthmüller, Efficient iterative schemes for ab

initio total-energy calculations using a plane-wave basis set, Phys. Rev. B 54, 11169 (1996).
